# Towards Electrotuneable Nanoplasmonic Fabry–Perot Interferometer

**DOI:** 10.1038/s41598-017-19011-4

**Published:** 2018-01-12

**Authors:** Hayley Weir, Joshua B. Edel, Alexei A. Kornyshev, Debabrata Sikdar

**Affiliations:** 1 Department of Chemistry, Imperial College London, London, SW7 2AZ UK; 2 Department of Chemistry, Stanford University, 333 Campus Drive, Stanford, CA 94305 USA; 3 Department of Electronics and Electrical Engineering, Indian Institute of Technology Guwahati, Guwahati, 781039 India

## Abstract

Directed voltage-controlled assembly and disassembly of plasmonic nanoparticles (NPs) at electrified solid–electrolyte interfaces (SEI) offer novel opportunities for the creation of tuneable optical devices. We apply this concept to propose a fast electrotuneable, NP-based Fabry–Perot (FP) interferometer, comprising two parallel transparent electrodes in aqueous electrolyte, which form the polarizable SEI for directed assembly–disassembly of negatively charged NPs. An FP cavity between two reflective NP-monolayers assembled at such interfaces can be formed or deconstructed under positive or negative polarization of the electrodes, respectively. The inter-NP spacing may be tuned *via* applied potential. Since the intensity, wavelength, and linewidth of the reflectivity peak depend on the NP packing density, the transmission spectrum of the system can thus be varied. A detailed theoretical model of the system’s optical response is presented, which shows excellent agreement with full-wave simulations. The tuning of the peak transmission wavelength and linewidth is investigated in detail. Design guidelines for such NP-based FP systems are established, where transmission characteristics can be electrotuned *in-situ*, without mechanically altering the cavity length.

## Introduction

Tuneable Fabry-Perot (FP) interferometers have been the subject of significant research owing to their use in many applications, such as optical fibre sensing, gas analysis, spectrometry, tuneable filters, chemical sensing, biosensing and refractive index measurements^1–8^.

An FP interferometer comprises two parallel reflective surfaces, which result in multiple beam interference of any light that enters into the cavity^9^. Only wavelengths that satisfy the constructive interference condition are transmitted, with the remaining light undergoing destructive interference. This results in sharp transmission peaks, whose wavelengths are determined by the cavity length.

Electrically tuneable FP interferometers, based on parallel reflector plates, allow tuning of the transmittance spectrum and have been realised through the use of liquid crystals, silicon and organic-inorganic hybrid methods, just to name a few^10–16^. There are numerous examples of tuneable, micromachined FP systems, however, in many cases these require expensive and complicated top-down fabrication procedures^17–20^. It is also common for these devices to demand large tuning potentials, causing them to be energetically expensive and increasing the risk of degradation of the device and sample over time. Therefore, an alternative bottom-up design may be interesting, in which the reflective properties of the cavity ‘mirrors’ can be altered with ultra-low voltage variation. This could improve the energy efficiency as well as response time.

Plasmonic nanoparticles (NPs) have previously been employed to enhance the transmission signal of the FP interferometers^21,22^ and achieve optical tuning, through the use of TiO_2_ NP thin film coating^23^, embedded Au NPs^24^, and hybrid NP-cavities^25,26^.

A property of NPs that has been the subject of over a decade of research is their ability to undergo directed assembly and disassembly at an electrochemical interface, triggered by the variation of applied potential^27,28^. This is achieved by functionalizing the NPs with charged ligands and exposing them to a polarisable interface. A positively charged interface will attract negatively charged NPs, resulting in the formation of a closely packed two dimensional NP layer. Such NP monolayer will reflect incident light, enacting a ‘mirror’. Conversely, a negatively charged interface will repel the NPs, causing them to remain dispersed in the bulk solution. In this configuration, incident light simply passes through the interface, enacting a ‘window’. Such an electrotuneable mirror-window platform, powered by controlled NP assembly, can be used to create electrotuneable, reversible optical devices with sub-volt tuning potentials. This has been experimentally achieved for electrochemical liquid|liquid interfaces^29^; work in our group is in progress on the structures based on transparent solid electrodes.

**Figure 1:**
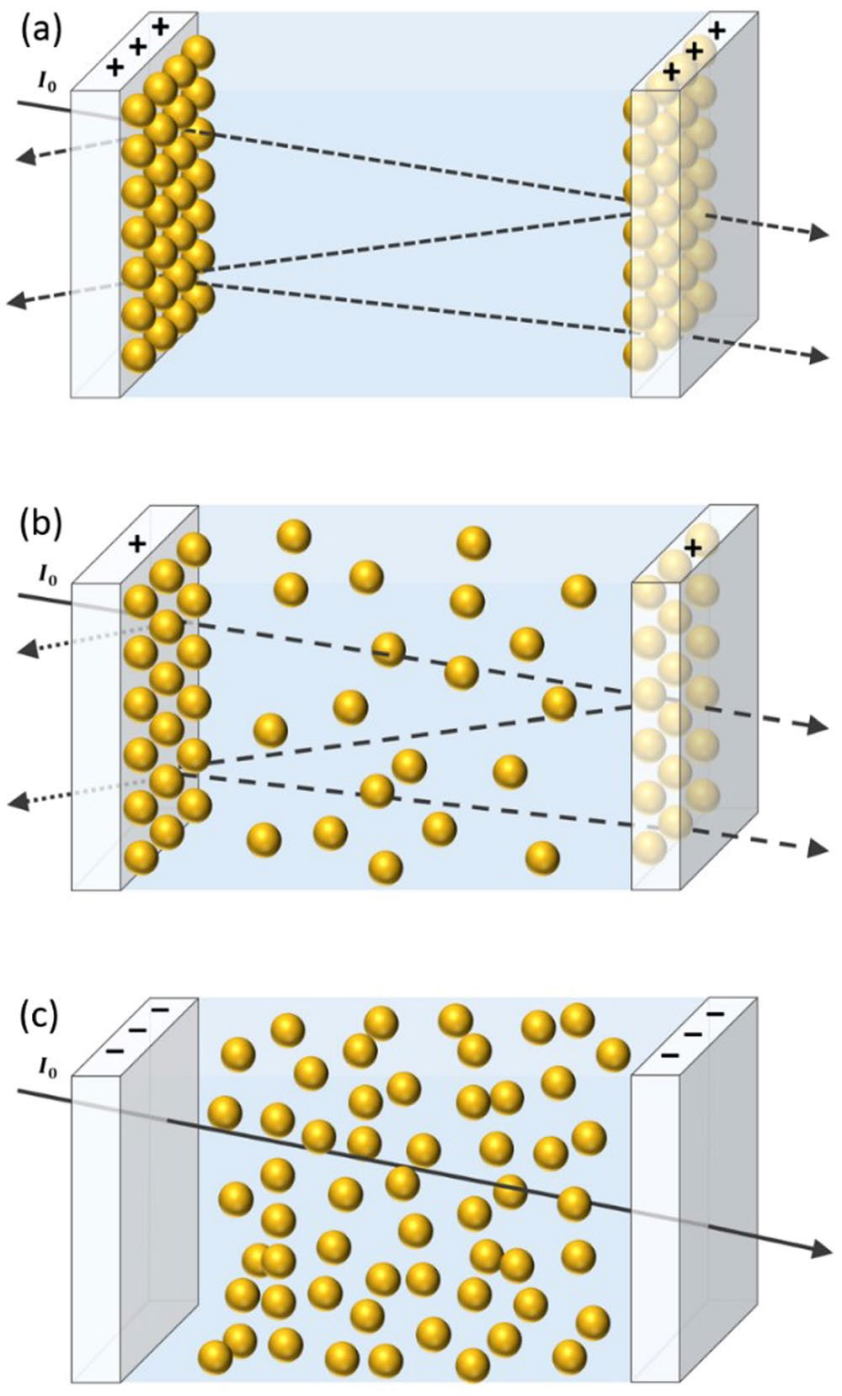
Schematic of an electrotuneable FP interferometer comprised of negatively charged NPs and two parallel ITO electrodes in aqueous electrolyte. Electrodes can be polarized with respect to a reference electrode, not shown. The particles are confined within the cavity volume by membranes, not shown, permeable for the electrolyte solution, but not the NPs. (**a**) For strong positive electrode polarisation, NPs assemble along the ITO-water interface in densely packed arrays to form an FP-cavity; (**b**) for weak positive electrode polarisation, sparse layers of NPs are formed with reduced plasmon coupling and reflectivity; (**c**) for negative electrode polarisation, NPs remain dispersed in the bulk and do not exhibit any significant absorption or scattering of light (due to absence of collective plasmon resonance), allowing almost-complete transmittance of the incident light with negligible losses within the cavity.

Here we propose an NP-based FP cavity comprising two parallel transparent electrodes, based e.g. on indium tin oxide (ITO) electrodes immersed in an aqueous electrolytic NP solution (other transparent conducting electrodes can be also considered, depending on their electrochemical stability, and if dealing with electrochemically less stable ITO, the latter should be protected by a thin conducting transparent layer of, say, titanium nitrate or graphene). Directed assembly of plasmonic NPs at the positively charged ITO-water interfaces leads to the formation of two NP mirrors, and hence an FP-cavity gets constructed. Since the linewidth and wavelength of the transmission peak are functions of NP-packing density, and hence based on the extent of positive polarisation of the electrodes, fast *in-situ* tuning of the transmittance spectrum can be achieved at a fixed cavity length. This is due to changes in the collective plasmon resonance and reflectivity of the NP mirrors^30–32^. Under sufficient negative electrode potential, complete dissociation of the NP layers is expected. This allows ~100% transmittance of incident light over the entire wavelength range, effectively ‘switching off’ the cavity. These different configurations and the path of incident light are illustrated in Fig. 1.

Such ‘bottom-up’ systems increase both the ease of fabrication and energy efficiency of tuning the optical response. Furthermore, an entirely new transmission spectrum may be obtained, without the need to change the physical set-up of the system. This can be achieved by simply replacing the NPs participating in cavity formation with NPs of alternative size, shape, or composition.

In this paper, we present the theoretical model for the proposed system, evaluate its transmission characteristics and tuneability, and assess the device’s viability for applications such as electrotuneable optical cavities and sensors.

## Methods

### Theoretical Model Formulation

**Figure 2:**
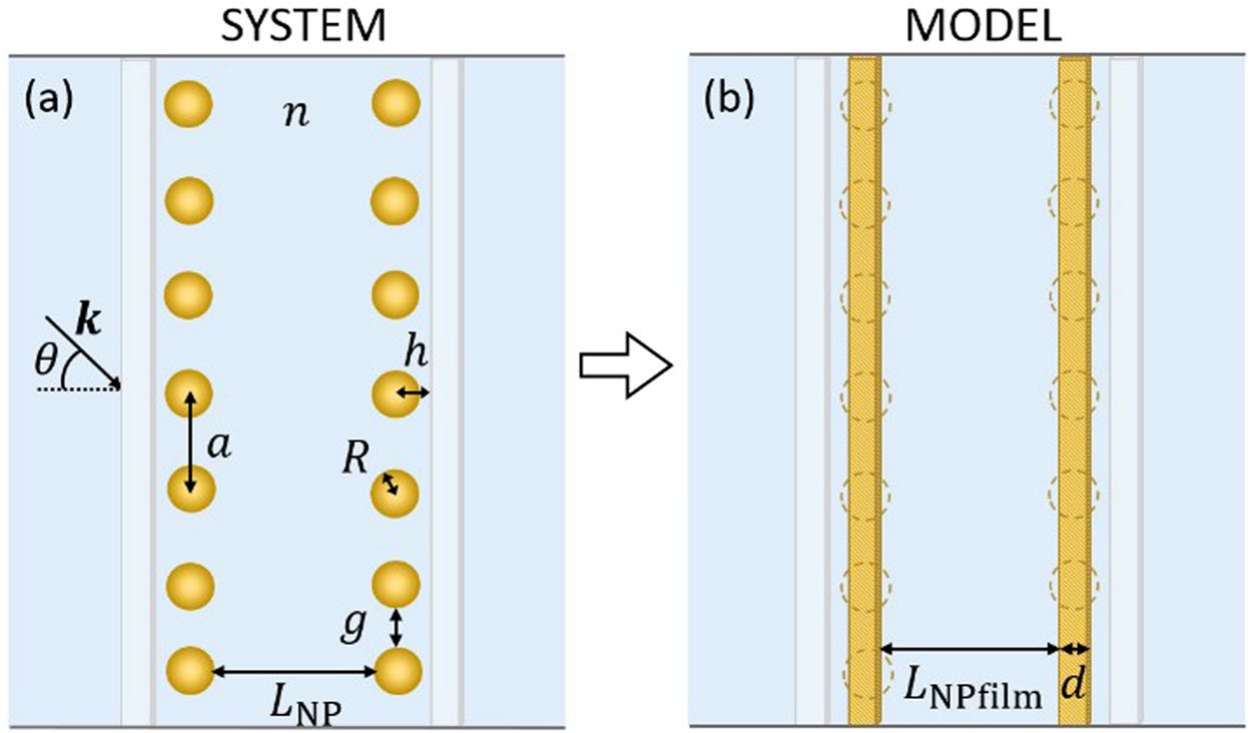
Theoretical model of the NP-based Fabry-Perot system. (**a**) Real system, consisting of NPs assembled along ITO electrodes in water. (**b**) Equivalent model consisting of two continuous pseudo-NP films along ITO electrodes.

We model the assembled 2D NP monolayers as uniform ‘pseudo-films’, as depicted in Fig. 2. The longitudinal and transversal effective dielectric permittivities of such a film for hexagonal close-packed NPs of radii, *R*, with equidistant inter-NP gaps, *g*, were obtained following refs^33,34^, as

1a $${{\epsilon }}_{{\rm{NPfilm}}}^{\parallel }(\omega )={{\epsilon }}_{{\rm{med}}}+\frac{8\pi }{\sqrt{3}{a}^{2}d}{\beta }_{\parallel }(\omega )$$

1b $${{\epsilon }}_{{\rm{NPfilm}}}^{\perp }(\omega )=\frac{1}{{{\epsilon }}_{{\rm{med}}}}+\frac{1}{{{\epsilon }}_{{\rm{med}}}^{2}}\frac{8\pi }{\sqrt{3}{a}^{2}d}{\beta }_{\perp }(\omega )$$

Here, $d=\frac{4\pi {R}^{3}}{3{a}^{2}}$ is the thickness of the film*; a* = 2*R* +* g* is the NP-lattice constant; *∈*_med_ is the permittivity of the surrounding liquid (here, water is considered in all calculations) and ${\beta }_{\parallel ,\perp }(\omega )$ are the effective quasi-static dipolar polarisabilities of the pseudo-film. It is important to note that the permittivity of such fictitious film is not equal to the permittivity of pure gold. Equation (1) uses the permittivity of pure gold obtained from a Drude-Lorentz model fit^32^ that closely matches the experimental data measured by Johnson and Christy^35^. In this study we considered 50 nm thick films as the ITO electrodes whose permittivity values were taken from ref.^32^. The gap between each NP and the electrode surface, when NPs assemble in form of a monolayer, is assumed to be 2 nm, which is typically the length of the NP-capping ligands.

The two NP monolayers are separated by the cavity length, *L*_NP_, which can be mapped onto a surface-to-surface length between two pseudo films, given by

2 $${L}_{{\rm{NPfilm}}}={L}_{{\rm{NP}}}+2R-d.$$

The reflectance, transmittance and absorption of the system were calculated using a multilayer Fresnel scheme. For that, the wave vectors and phase shifts were calculated for each layer, and the reflection and transmission coefficients for each interface were determined (for details see refs^33,34^). This *effective medium theory* (EMT) is valid for NPs of radius less than 50 nm, as, for larger ones, the quasi-static dipolar approximation breaks down.

### Full-wave simulation model

The full-wave simulations of the proposed gold-nanoparticles-based FP cavity was conducted using the RF module of the commercially available FEM software—COMSOL Multiphysics^®^. Frequency domain solver was deployed for the simulation studies. We assumed gold nanospheres to form a monolayer at the interfaces, which can be closely estimated as a hexagonal two-dimensional array. A unit cell was designed, which extended in both lateral dimensions using periodic boundary conditions to emulate monolayers assembled on each of the ITO electrodes to form the FP cavity. In simulation studies we do not consider extra NPs dispersed in the bulk. In all simulations we considered mesh-size to be extremely-fine, where the maximum and the minimum element size was chosen to be λ/10 (where λ is the wavelength of light in that medium) and 1.5 nm, respectively. With such meshing, all structural details of the system can be accurately incorporated in the full-wave simulations.

## Results and Discussion

**Figure 3:**
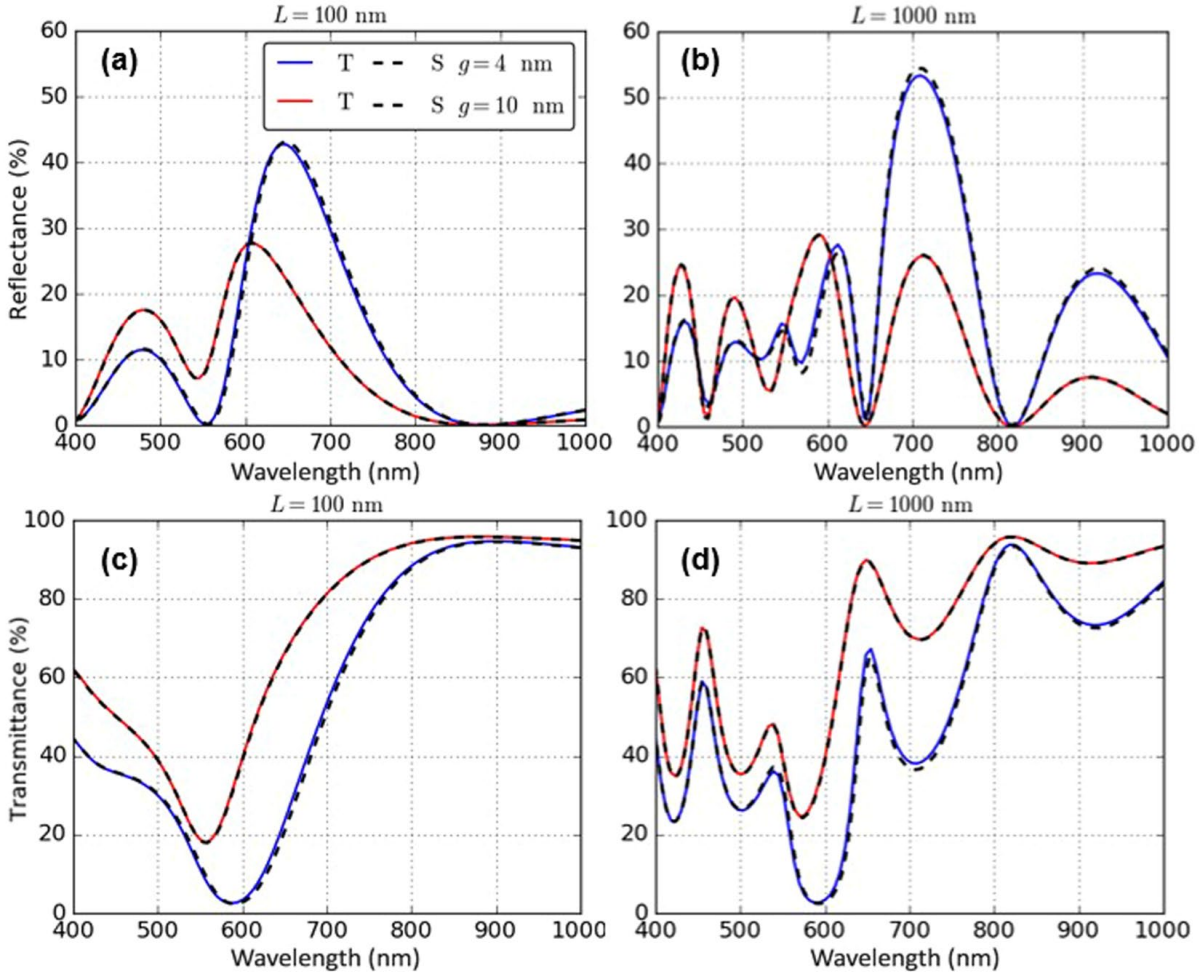
Comparison of the multi-layer Fresnel scheme theory, T (solid line) with full-wave simulations, S (dotted line) for calculation of the (**a**,**b**) reflectance and (**c**,**d**) transmittance spectra of a Fabry-Perot cavity comprised of 10 nm radius NPs. Cavity lengths: (**a**,**c**) 100 nm, (**b**,**d**) 1000 nm; Inter-NP gaps: 4 nm, 10 nm.

Figure 3 shows a comparison between the reflectance and transmittance spectra of the proposed system calculated with EMT and COMSOL Multiphysics^®^ full wave simulations for two extreme cases of short (*L* = 100 nm) and long (*L* = 1000 nm) cavity lengths. The excellent coherence between the two methods gives us confidence that the EMT-model can be applied to describe optical characteristics of the proposed plasmonic NP-based FP-cavity.

**Figure 4:**
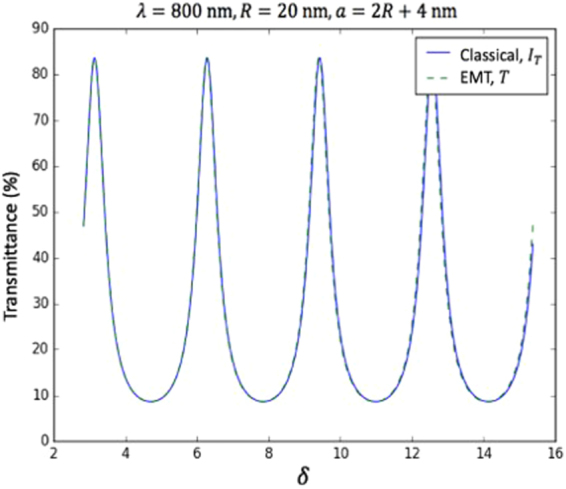
Comparison between transmittance against phase delay (*δ*) calculated with the multilayer Fresnel scheme theory (*T*) and with the classical Fabry-Perot theory (*I*_*T*_) for a Fabry-Perot system constructed from 20 nm radius NPs.

EMT results are compared with the classical FP theory. The latter relies on the fact that a wavelength (*λ*) will be transmitted if the cavity length is equal to a multiple of $\frac{\lambda }{2}$. When this condition is satisfied, the wall of the cavity coincides with a node of the light wave, allowing constructive interference, and hence generating a standing wave. The transmitted intensity of an absorbing FP cavity can be calculated as^9^

3 $${I}_{{\rm{T}}}=\frac{1}{1+F\,{\sin }^{2}\frac{\delta }{2}}{K}^{2}$$

where $F=\frac{4{r}^{2}}{1-{r}^{2}}$ is the coefficient of finesse, $K=\frac{1-A-{r}^{2}}{1-{r}^{2}}$, *r* is the reflection of one mirror, and *δ* is the phase difference between each adjacent transmitted wave given by^9^

4 $$\delta =\frac{4\pi nd\,\cos \,\theta }{\lambda }-2\text{arg}(r)$$

where *n* is the refractive index of the cavity medium, and *θ* is the angle of incident light. Calculating *r* from our EMT, we can compare the transmission calculated from the classical result with direct EMT calculation of the full system. Figure 4 illustrates the perfect match between the two methods, thereby demonstrating the FP behaviour of the NP-based device.

Note that for electrotuneable systems with repeated cycles of assembly/disassembly of NPs at polarized interfaces, it is a common practise to have some excess amount of NPs over those exactly required to cover the interface^28^. That would account for any loss of NPs along the walls of the device during its operation, to ensure a sufficient NPs available to form the monolayer at the interface. Our previous experience with a liquid|liquid cell has shown that the amount of NPs about twice the one needed to fully cover the interface is usually enough. Similar precautionary measure may also be taken for our proposed device. But in a liquid|liquid cell, the functional part of the system was the liquid|liquid interface, but not the walls of the container. In the Fabry-Perot resonator, the front flat walls are the functional parts, and the losses could be only due to adsorption to side walls of much smaller area. Taking 50% excess of NPs would therefore be perfectly sufficient, as the surface area of the side walls will never exceed 50% of the front walls. Therefore, in order to closely model a practical system, besides NPs forming monolayers on the electrodes, the excess NPs that remain in the bulk within the cavity must also be accounted for in the calculations. Overall the total number of NPs distributed between adsorbed and bulk states, *N*_tot_ must remain constant. For dense NP assemblies, the excess number of NPs will remain in the aqueous phase, with this number increasing if the NP layers are sparsely packed. This could affect the dielectric properties of the solution. It is therefore necessary to recalculate the properties of the cavity medium for each monolayer packing density. As a result, the effective permittivity of the cavity medium is re-calculated, each time, using the Maxwell-Garnett formula^36^ given by

5 $${{\epsilon }}_{{\rm{med}}}^{{\rm{MG}}}={{\epsilon }}_{{{\rm{H}}}_{2}0}\frac{2f({{\epsilon }}_{{\rm{NP}}}-{{\epsilon }}_{{{\rm{H}}}_{2}0})+{{\epsilon }}_{{\rm{NP}}}+2{{\epsilon }}_{{{\rm{H}}}_{2}0}\,}{f({{\epsilon }}_{{{\rm{H}}}_{2}0}-{{\epsilon }}_{{\rm{NP}}})+{{\epsilon }}_{{\rm{NP}}}+2{{\epsilon }}_{{{\rm{H}}}_{2}0}}.$$

Here ${{\epsilon }}_{{\rm{N}}\mathrm{P}}$ and ${{\epsilon }}_{{{\rm{H}}}_{2}0}$ are the dielectric permittivities of the individual NPs and of water, respectively; *f* is the volume fraction of NPs in the cavity, given by

6 $$f=\frac{({N}_{{\rm{tot}}}-2{N}_{{\rm{mono}}}){V}_{{\rm{NP}}}}{w\times h\times L}$$

where *w*, *h* and *L* are the width, height and length of the cavity formed by the two ITO electrodes, respectively. *N*_tot_ is the number of NPs required to exactly cover the surface area of both electrodes. It is a common practice in experiments to take some excess NPs. We consider a 50% excess of NPs in our calculations, thus ${N}_{{\rm{tot}}}=\frac{2\times w\times h}{4\pi {R}^{2}}\times 1.5$. Note that the methodology described here can be readily adapted to estimate the optical performance based on any number of excess NPs.

The number of NPs assembled forming each monolayer, with inter-NP gap of *g*, at the water-ITO interfaces is ${N}_{{\rm{mono}}}=\frac{2\times w\times h}{4\pi {(R+\frac{g}{2})}^{2}}$ and the volume of one NP is ${V}_{{\rm{NP}}}=\frac{4}{3}\pi {R}^{3}$.

**Figure 5:**
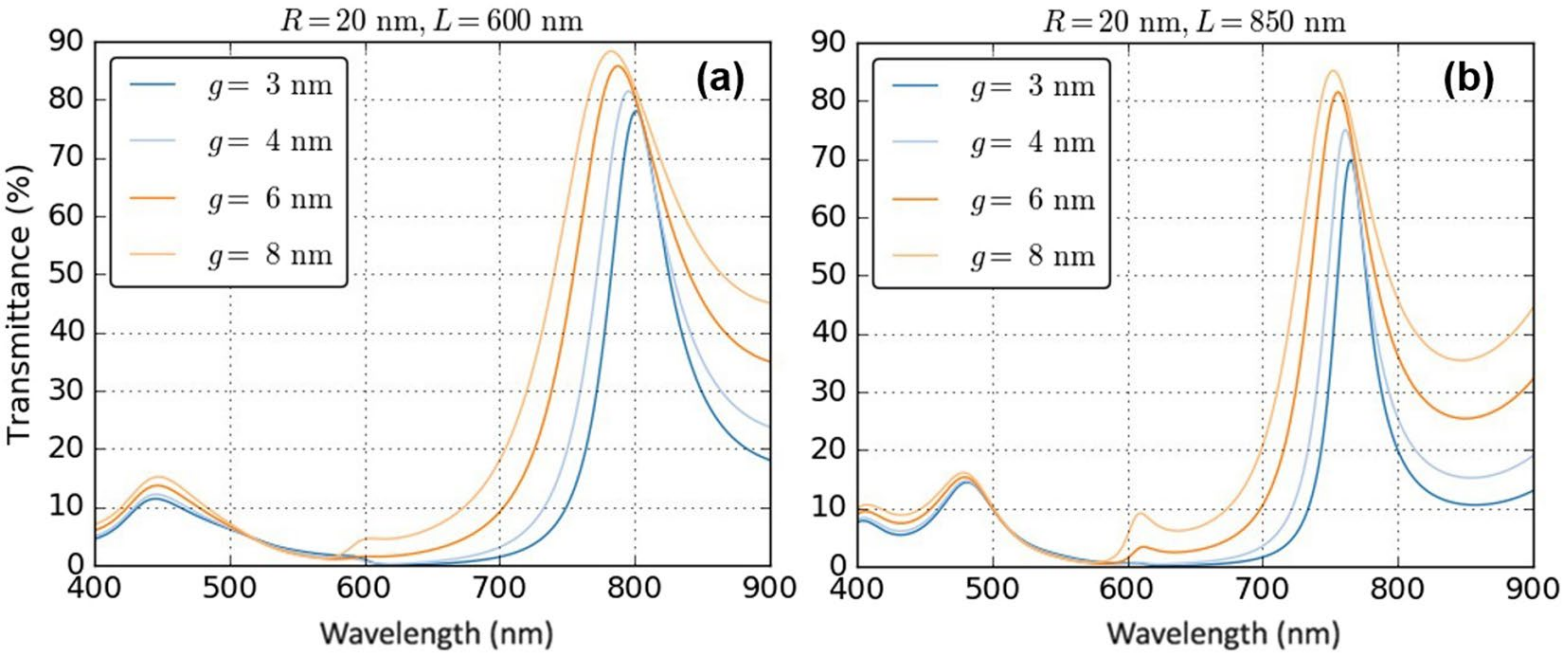
Demonstration of electrotuning of the transmittance peak in terms of changes in the inter-NP gap: for NPs with 20 nm radius, cavity length of (**a**) 600 nm and (**b**) 850 nm.

The NP-based FP system allows the transmittance spectra to be electrotuned *in-situ*. By changing the inter-NP gap, both the peak transmission wavelength and finesse can be varied dramatically. Desired spectral characteristics can be obtained by the varying electrode polarization, which affects, as described, the NP packing density. Densely packed monolayers result in highly reflective mirrors, giving rise to sharp, red-shifted transmission peaks, whereas sparse packing results in low reflection, giving broad, blue-shifted transmission peaks, *c.f.* Fig. 5. A red-shift is observed with decreasing inter-NP gap due to the increase in the strength of bonding-type plasmonic coupling between the NPs.

The proposed platform offers many advantages including the possibility to quickly ‘switch-off’ the cavity, *via* application of a negative potential to the electrodes. For example, the speed of the assembly and disassembly can be calculated using the simple relation

7 $$\tau \sim \frac{{l}^{2}}{{D}_{{\rm{NP}}}}.$$

where *D*_NP_ is the NP diffusion coefficient, and *l* is the characteristic length^29^:

8 $$l \sim \frac{1}{{c}_{{\rm{NP}}}\xi \pi {R}^{2}}\,\,({\rm{assembly}}),\,l \sim 10R\,({\rm{disassembly}})$$

where *c*_NP_ is the concentration of NPs, *ξ* is the NP packing factor at the interface, and *R* is the NP radius.

Using identical parameters to those as shown in Fig. 5(a), the time of assembly and disassembly can be approximated to be as little as 13 ms and 1 ms, respectively. Practically such as cavity can be easily realized using microfabrication and sacrificial layer techniques^37^. Albeit the transparent electrode would have to be deposited using methods such as e-beam evaporation or atomic layer deposition and would have to be resistant to the sacrificial layer (*e.g.* Chrome) which can be etched away either electrochemically or *via* solvent. The thickness of the sacrificial layer would govern the length of the cavity. This strategy could yield cavity lengths of anywhere from 10’s–100’s of nm. Very small length will not be needed, because the NPs will have no room to leave the surface, but the range of few 100’s of nm will be practical. Alternatively, another strategy could involve the deposition of a photoresist such as SU-8 onto a conductive substrate, a second conductive substrate could then be bonded on top to form a sandwich. SU-8 can be patterned to any desired shape using photolithography and wet etching techniques with the length of the cavity convened by the original resist thickness.

## Conclusion

We proposed an original design of tuneable electrochemical Fabry–Perot cell that can be used for fast *in-situ* electrotuning of the optical transmittance spectra.

Besides tuneablity through voltage variation, the design will also allow the cavity properties to be varied by altering NP characteristics (their size, shape, and composition), types of ligands, charge of ligands through changing the solution pH, and interaction between NPs *via* changing electrolyte concentration, while keeping the physical set-up of the system unaltered.

Such a ‘bottom–up’ approach of designing tuneable optical devices would benefit from the ease of fabrication, programmability, and energy efficiency of tuning of the optical responses. Guidelines for designing these practical systems are established. The presented principles and theoretical analysis of plasmonic NP-based FP-cavities will navigate the construction and optimization of the properties of such novel electrotuneable-optical devices.
